# Co-registration Comparison of On-Scalp Magnetoencephalography and Magnetic Resonance Imaging

**DOI:** 10.3389/fnins.2021.706785

**Published:** 2021-08-13

**Authors:** Fuzhi Cao, Nan An, Weinan Xu, Wenli Wang, Yanfei Yang, Min Xiang, Yang Gao, Xiaolin Ning

**Affiliations:** ^1^School of Instrumentation and Optoelectronic Engineering, Beihang University, Beijing, China; ^2^Hangzhou Innovation Institute, Beihang University, Hangzhou, China; ^3^Research Institute for Frontier Science, Beihang University, Beijing, China; ^4^Beijing Academy of Quantum Information Sciences, Beijing, China

**Keywords:** magnetoencephalography, on-scalp MEG, co-registration, reference phantom, laser scanner

## Abstract

Magnetoencephalography (MEG) can non-invasively measure the electromagnetic activity of the brain. A new type of MEG, on-scalp MEG, has attracted the attention of researchers recently. Compared to the conventional SQUID-MEG, on-scalp MEG constructed with optically pumped magnetometers is wearable and has a high signal-to-noise ratio. While the co-registration between MEG and magnetic resonance imaging (MRI) significantly influences the source localization accuracy, co-registration error requires assessment, and quantification. Recent studies have evaluated the co-registration error of on-scalp MEG mainly based on the surface fit error or the repeatability error of different measurements, which do not reflect the true co-registration error. In this study, a three-dimensional-printed reference phantom was constructed to provide the ground truth of MEG sensor locations and orientations relative to MRI. The co-registration performances of commonly used three devices—electromagnetic digitization system, structured-light scanner, and laser scanner—were compared and quantified by the indices of final co-registration errors in the reference phantom and human experiments. Furthermore, the influence of the co-registration error on the performance of source localization was analyzed via simulations. The laser scanner had the best co-registration accuracy (rotation error of 0.23° and translation error of 0.76 mm based on the phantom experiment), whereas the structured-light scanner had the best cost performance. The results of this study provide recommendations and precautions for researchers regarding selecting and using an appropriate device for the co-registration of on-scalp MEG and MRI.

## Introduction

Magnetoencephalography (MEG) can directly measure the external magnetic field generated from pyramidal neurons synchronously activated in the brain. MEG has been widely used in clinical and neuroscience studies (Hansen et al., 2010; Baillet, 2017). Low-Tc superconducting quantum interference devices (SQUID)-MEG has become a reliable technology after 30 years of development. However, it operates at the temperature of liquid helium (4 K), resulting in expensive maintenance costs. In addition, the requirement for temperature insulation between sensors and the scalp increases the distance between them to approximately 2 cm (Zetter et al., 2019; Gu et al., 2021), reducing the signal-to-noise ratio (SNR) of the brain signal. New technologies have emerged to overcome the low SNR, including optically pumped magnetometers (OPMs) (Tierney et al., 2018; Vivekananda et al., 2020) and high-temperature SQUIDs (Pfeiffer et al., 2019; Schneiderman et al., 2019), which can be placed very close to the scalp. Among these, on-scalp MEG constructed with OPMs increases the SNR by approximately 3–5-fold (Tierney et al., 2020). By customizing a personalized helmet, OPM-based on-scalp MEG is wearable and suitable for people with different head circumferences, especially developing children (Boto et al., 2018, 2021; Hill et al., 2019).

Magnetoencephalography helps researchers to localize the origins of neuromagnetic signals. Magnetic source imaging requires a cortical surface-based model derived from magnetic resonance imaging (MRI). However, the MEG and MRI data are obtained from different devices; hence, establishing the accurate position and orientation of MEG sensors relative to MRI (i.e., co-registration) is required. The source localization accuracy of MEG depends heavily on the co-registration accuracy (Chella et al., 2019). Precise estimation of brain anatomies such as the cortical column orientation (Bonaiuto et al., 2020) and the spatial extent of neuronal activation (Hillebrand and Barnes, 2011) will improve the source localization; however, it requires accurate co-registration. In addition, accurate co-registration is needed for the further development of MEG, such as for studying non-invasive laminar inference (Troebinger et al., 2014; Bonaiuto et al., 2018) and detecting the amygdala and hippocampus (Tzovara et al., 2019).

The co-registration of SQUID-MEG is usually accomplished using head position indicator (HPI) coils and an electromagnetic digitization system (Ahlfors and Ilmoniemi, 1989; Bardouille et al., 2012; Vema Krishna Murthy et al., 2014; Zetter et al., 2019). The co-registration of on-scalp MEG differs from that of the traditional SQUID-MEG due to its customized and flexible helmet configuration. There are two types of helmets for on-scalp MEG—flexible and rigid. The flexible helmet is lighter than the rigid helmet and can be positioned closer to the scalp; however, the sensors are prone to relative displacements, increasing random errors in sensor position and orientation. These random errors have a greater impact on the source localization accuracy compared to the systematic errors of the rigid helmet (Hill et al., 2020). Therefore, Hill et al. suggest the use of a rigid helmet. The commonly used co-registration devices for on-scalp MEG include the electromagnetic digitization system (Polhemus) (Hill et al., 2019), structured-light scanner (Zetter et al., 2019), and laser scanner (Gu et al., 2021). The co-registration accuracies of these devices require comprehensive evaluation. Zetter et al. (2019) and Hill et al. (2020) used the structured-light scanner to preliminarily study the co-registration accuracy of on-scalp MEG and MRI. Zetter et al. used surface fit error and reproducibility error of different measurements to evaluate co-registration errors. However, the surface fit error is one of the RMS type errors. It has been shown that the RMS type errors are uncorrelated with the co-registration error (Fitzpatrick, 2009; Sonntag et al., 2018). Hill et al. calculated the co-registration accuracy using the average distance deviation between the co-registered average sensor positions of all groups and the positions of each group. This deviation is more likely to represent the measurement of repeatability than the actual co-registration error. In addition, Pfeiffer et al. (2018, 2020) introduced a novel approach for localizing on-scalp MEG sensors using multiple localization coils and they analyzed the sensor location error by the random errors and systematic errors mainly come from the sensor noise, the coil position error, and the head movement.

The present study used a reference phantom as the ground truth for sensor locations and orientations relative to MRI and comprehensively compared the co-registration of on-scalp MEG and MRI among three devices (Polhemus, the structured-light scanner, and the laser scanner). The co-registration errors of different devices were analyzed in both the reference phantom and human experiments. In addition, we analyzed the influence of co-registration error on source localization in simulations. We have also provided suggestions and precautions for the co-registration of on-scalp MEG and MRI.

## Materials and Methods

### Helmet and Coordinate System

**FIGURE 1:**
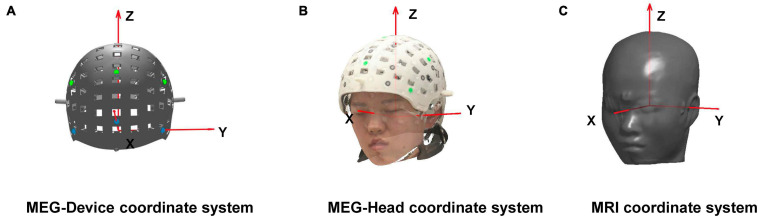
**(A)** The magnetoencephalography (MEG)-Device coordinate system is defined during designing the helmet. Here, the origin of the MEG-Device coordinates is the midpoint of the left and right blue markers. The X-axis points to the front blue marker, and the Y-axis points to the left blue marker. **(B)** The MEG-Head coordinate system is defined based on the nasion point, left and right pre-auricular points, which are chosen in the optical scanning or digital results. The X-axis points to the nasion point when the Y-axis points to the left ear point. **(C)** The magnetic resonance imaging (MRI) coordinate system is defined based on the three fiducial points: nasion point, left and right pre-auricular points, which are marked on the MRI slices. The origin of the MRI coordinates is the midpoint of the left and right pre-auricular points, and the X-axis points to the nasion point when the Y-axis points to the left pre-auricular point. All coordinate systems follow the right-hand rule.

We designed a rigid helmet with 85 sensor slots. The sensor positions and orientations in the helmet coordinate system were determined during helmet design. Each sensor position was defined as the center point of the bottom surface of each slot, and each sensor orientation was defined as the vector starting from the center point of the bottom surface to that of the top surface of each slot. To ensure that the helmet was fixed relative to the head during measurements, three-bolt locking structures were designed for the helmet. As shown in Figure 1A, six reference points used for the co-registration were designed at symmetrical positions on the outer surface of the helmet. After the design phase, the helmet was printed using DSM 8000 resin on a Lite 600 three-dimensional (3D) printer (UnionTech Inc., Songjiang, SH, China) with an accuracy of ±0.2 mm.^1^

The purpose of co-registration is to obtain the sensor positions and orientations relative to MRI. For on-scalp MEG, co-registration involves a two-step transformation between the three coordinate systems, as shown in Figure 1. *Transform 1* involves a transformation from the MEG-Device coordinate system (Figure 1A) to the MEG-Head coordinate system (Figure 1B). *Transform 2* involves a transformation from the MEG-Head coordinate system to the MRI coordinate system (Figure 1C).

### Co-registration Devices and Methods

#### Electromagnetic Digitization System (Polhemus)

The most commonly used digital device for MEG is the electromagnetic digitization system (Polhemus Inc., Colchester, VT, United States), which uses an alternating current electromagnetic transmitter and receiver to digitize the positions of spots in space. The device has a static position accuracy of 0.8 mm and an orientation accuracy of 0.15° when receivers are located within 76 cm of the transmitter.^2^

**FIGURE 2:**
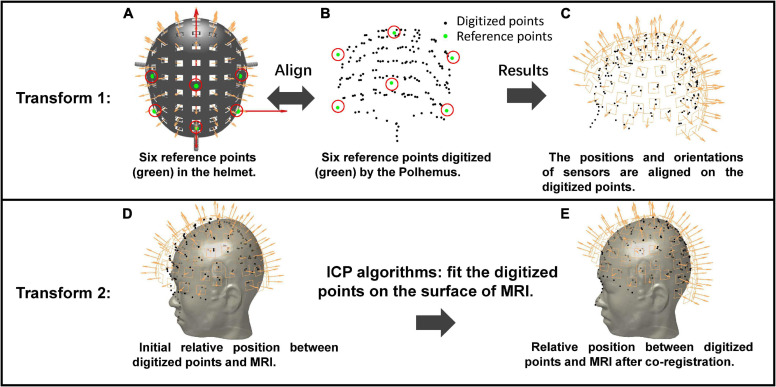
The operation process of the co-registration using Polhemus. Transform 1: Align the positions of the six reference points between the MEG-Device coordinate system **(A)** and the MEG-Head coordinate system **(B)** to let the sensor positions and orientations be aligned on the digitized points **(C)**. Transform 2: Use the ICP algorithm to fit the digitized points onto the MRI. **(D,E)** The initial relative positions between digitized points and MRI and relative positions after ICP, respectively.

The co-registration method using the Polhemus is illustrated in Figure 2. The initial sensor positions and orientations are in the MEG-Device coordinate system (Figure 2A). The first step is to align the six reference points of the helmet with the recorded digitized points (green circles in Figure 2B). The alignment results are shown in Figure 2C, indicating that the sensor position and orientation are transformed from the MEG-Device coordinate system to the MEG-Head coordinate system. The second transformation involves the segmentation of MRI data of the participant (the gray human head in Figure 2D) using Freesurfer (Fischl, 2012). The segmented scalp is initially in MRI coordinate system and not aligned with digital points, as shown in Figure 2D. Therefore, the next step is to use the iterative closest point (ICP) algorithm (Besl and McKay, 1992) to match the digitized points (black circles in Figure 2C) with the outer surface of the scalp (the gray human head in Figure 2D). The sensor positions and orientations are then transformed into the MRI coordinate system. The final results of the co-registration between MEG and MRI are shown in Figure 2E.

#### Structured-Light Scanner

A consumer-grade structured-light scanner (Occipital Inc., San Francisco, CA, United States) was used, which can provide rapid 3D scanning of objects and real-time display of the results. The accuracy of the structured-light scanner is 0.8 mm under the typical working distance of 50 cm.^3^

**FIGURE 3:**
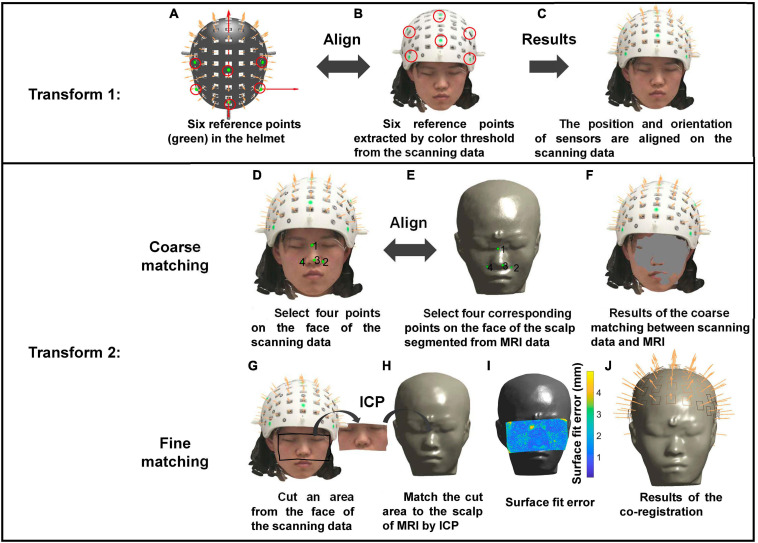
The operation process of the co-registration using the structured-light scanner. Transform 1: Align six reference points on the helmet **(A)** and those extracted by color threshold from the scanning data **(B)** to let the positions and orientations of sensors be aligned on the scanning data **(C)**. Transform 2: Perform coarse matching **(D–F)** and fine matching **(G–I)** successively to complete the co-registration. **(J)** The co-registration results.

The co-registration method based on the structured-light scanner is illustrated in Figure 3. The structured-light scanner can provide scanning images with color information. Thus, the color threshold method can be used to extract the six reference points from the scanning data. The first transformation is performed by aligning the reference points extracted from the scanning data (Figure 3B) with those on the helmet (Figure 3A). The second transformation includes two steps, coarse and fine matching. First, coarse matching is achieved by aligning the four corresponding points separately selected from the scanning data and the face derived from MRI. Then, fine matching is performed using the ICP algorithm to match the point cloud of the face from the scanning data and that from the MRI. Previous studies have shown that sufficient matching accuracy can be obtained by selecting a part of the face (Koessler et al., 2011; Bardouille et al., 2012). Therefore, intercepting part of the face area improves the computational efficiency while providing satisfactory accuracy (Figure 3I).

#### Laser Scanner

An industrial-grade handheld 3D laser scanner (HSCAN Prince 775, Scantech Inc., Hangzhou, ZJ, China) with an accuracy of 0.03 mm under a working distance of 30 cm was used to perform the co-registration.^4^

**FIGURE 4:**
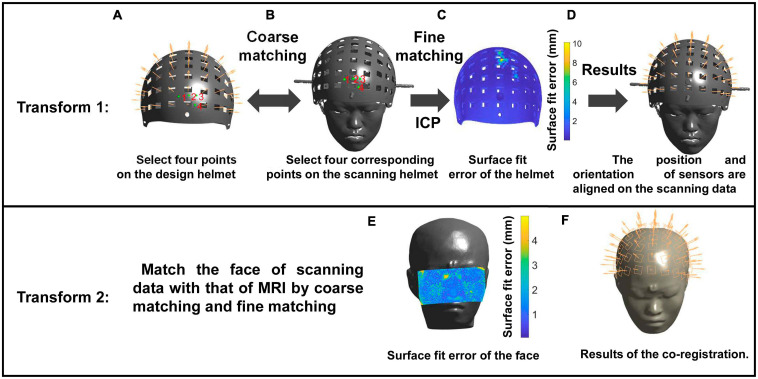
The operation process of the co-registration method using the structured-light scanner. Transform 1: Match the helmet of the design data with the scanning data through coarse **(A,B)** and fine matching **(C)** to align sensors on the digitized points **(D)**. Transform 2: Match the cut face of scanning data with the MRI data to complete the co-registration. **(E,F)** The surface fit error of the face and the co-registration results, respectively.

The co-registration method based on the laser scanner is illustrated in Figure 4. The laser scanner is used to obtain a 3D superfine stereo image without color information. Thus, the first transformation is performed by matching the 3D data of the designed helmet with that of the scanning helmet. Four corresponding points are selected from the helmet (Figure 4A) and optical scanning results (Figure 4B) and aligned for coarse matching. Then, fine matching is performed using the ICP algorithm for transformation from the MEG-Device coordinate system to the MEG-Head coordinate system. The matching results are shown in Figure 4D. For the second transformation, the same method as that used for the structured-light scanner is followed.

### Experiment

We constructed a reference phantom to compare the performance of the three above-mentioned devices and performed experiments using this reference phantom as well as a human participant. We then evaluated the effects of the co-registration errors of the three devices on the source localization accuracy via simulations.

#### Reference Phantom

**FIGURE 5:**
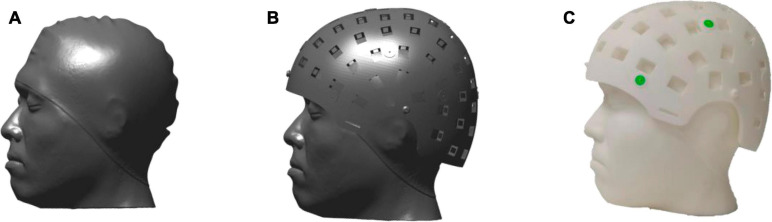
**(A)** The head of the subject scanned by the laser scanner. **(B)** Put the designed helmet on the scanning head. **(C)** The reference phantom made 3D printed.

The reference phantom was constructed as follows: (1) the laser scanner was used to scan and obtain the 3D structure of a participant wearing a swimming cap covering the hair (Figure 5A). The scanned image of the head was regarded as pseudo MRI. (2) The scanning image was imported to 3D software, and the designed helmet was placed on it (Figure 5B). Thus, the sensor positions and orientations relative to MRI were known. (3) The 3D structure composed of the scanning results and the helmet was 3D printed using the Lite 600 system with DSM 8000 resin with an accuracy of ±0.2 mm to produce the reference phantom (Figure 5C).

#### Experiment Protocol

The reference phantom and human experiments were performed in the afternoon. For the human experiment, data were obtained from a healthy 24-year-old woman. The research protocol was approved by the Ethical Committee of Beihang University, and written informed consent was obtained from the participant.

During data acquisition, the phantom was fixed at the same place for all measurements, and the participant was asked to remain as still as possible. The experiments started when all three devices were prepared. For each scanned object, each device digitized or scanned the object five times successively.

##### Polhemus

The transmitter was fixed to a wooded plank to avoid magnetic interference. A digital pencil and reference receiver were used in the experiment. The digital pencil allows users to quickly capture 3D data points. The reference receiver was fixed to the object. The recorded position and orientation of the reference receiver were used to calculate the rotation and translation of the object to correct the displacement error on the digital pencil caused by the movement of the object during data acquisition. In the digitization process, the distance between the transmitter and the digitized object was maintained within the working distance (76 cm) of the Polhemus. The reference points (Figure 2A) were first digitized twice, and then a large number of (more than 200) points on the object were digitized using the digital pencil. The average time required for single digitization was 3 min 44 s.

##### Structured-light scanner

The structured-light scanner was used in conjunction with an iPad and was calibrated before scanning. During the scanning process, the scanner was kept within the working distance (50 cm) from the scanned object and moved slowly to ensure that the 3D measurements were constantly integrated. The scanner automatically aligned consecutive image frames to reduce the effect of object movement. The average scanning time was 3 min. To obtain clearer results, the scanning results were further reconstructed using Skanect software.^5^

##### Laser scanner

The reflective targets were affixed to the object before scanning to function as positioning features on the shape. The scanner simultaneously scanned the 3D surface and tracked the movement of the object through these positioning features. The scanner did not need to integrate 3D measurements during scanning. Thus, its scanning time was shorter than that of the structured-light scanner. In our experiments, its average scanning time was 1 min 30 s.

After collecting the data, the corresponding co-registration method of each device, as summarized in Table 1, was used to obtain the position and orientation of the sensors relative to MRI in each experiment. Thus, MRI data were needed for co-registration. The phantom experiment used pseudo MRI data, while the human experiment used T1-weighted MRI data of the participant acquired on a 3-T scanner (Siemens Medical Solutions, Erlangen, Germany) using the following parameters: TR, 2,200 ms; TE, 3.37 ms; TI, 1,050 ms; FA, 7°; FOV, 256 × 256 mm; and voxel size, 1.0 × 1.0 × 1.0 mm^3^. The MRI data were segmented using Freesurfer (Fischl, 2012) to obtain a scalp surface with 50,000 vertices.

**TABLE 1:** Devices used and the corresponding co-registration methods.

**Device**	**Transform 1**	**Transform 2**
Polhemus	Align by reference points	Align by digital points (ICP)
Structured-light scanner	Align by reference points (color threshold)	Align by scanning cloud points of the face (ICP)
Laser scanner	Align by scanning cloud points of the helmet (ICP)	

#### Quantitative Metrics

Quantitative metrics were calculated to evaluate the performance of the three devices. The “known” position and orientation of each sensor relative to MRI are denoted as *r*_*m*_ and *v*_*m*_, respectively, while the co-registered position and orientation are denoted as $r_{m,n}^{\prime }$ and $v_{m,n}^{\prime }$ (*m* = 1, 2,…,*N*_*c*_,*n* =  1, 2,…,*N*_*p*_), respectively, where *m* represents the *m*-th sensor and *n* represents the *n*-th measurement. The number of sensors was *N*_*c*_ = 85, and the measurement times were *N*_*p*_ = 5 in our experiments.

##### Surface fit error

For on-scalp MEG, the co-registration included two-step transformations. The surface fit error was used to evaluate the matching error of each transformation. It should be noted that the surface fit error is uncorrelated with the co-registration error and it is just used as an index to demonstrate the quality of ICP fits. We used *A* = [*a*_1_,*a*_2_,…,*a*_*p*_] and *B* = [*b*_1_,*b*_2_,…,*b*_*q*_] to denote the two sets of point clouds that were matched. The surface fit error ε_*fit*_ is their average Euclidean distance, which is expressed as

(1) $$\begin{array}{c} \varepsilon_{f\,i\,t}=\frac{1}{p}\,\sum_{i=1}^{p}\min_{j=1,2,\ldots ,q}{\left|a_{i}-b_{j}\right|}_{ℱ} \end{array}$$

where |⋅|_ℱ_ is the *F*-norm. For the Polhemus, ε_*fit*_ of transform 1 was not calculated due to the lack of point cloud of the helmet. Therefore, the ε_*fit*_ of the Polhemus could only be evaluated from the second transformation, which was calculated between the registered digitized points and the scalp from MRI. For the two types of optical scanners, the surface fit errors of two transformations could be calculated. After matching, the ε_*fit*_ of the first transformation was calculated between the designed helmet and the scanning helmet, while the ε_*fit*_ of the second transformation was calculated between the scalp from MRI and the scanning face.

##### Repeatability error

Without the “ground truth” of the sensor positions and orientations, previous studies used the repeatability error of different measurements to represent the co-registration error (Zetter et al., 2019; Hill et al., 2020). In this study, we calculated this value from five measurement results. The repeatability error is defined as the average deviation of the values of each group from the average values of the five groups. Here, we calculated the repeatability errors of estimated transformation parameters (rotation and translation) as well as co-registered sensor location and orientation. For the *n*-th group data, the original sensor positions and orientations are transformed from the MEG-Device coordinate system to the MRI coordinate system via a translation vector *s*_*n*_ and a rotation quaternion *q*_*n*_ where *q* = *q*_0_ + *q*_1_*i* + *q*_2_*j* + *q*_3_*k* is a unit quaternion. The co-registered position and orientation of the *m*-th sensor are $r_{m,n}^{\prime }$ and $v_{m,n}^{\prime }$. We calculated the average translation for multiple measurements by $\bar{s}=\sum_{n=1}^{N_{p}}s_{n}/N_{p}$ and the average rotation by an average quaternion solution $\bar{q}=\sum_{n=1}^{N_{p}}q_{n}\,/\,\left|\sum_{n=1}^{N_{p}}q_{n}\right|$ (Gramkow, 2001). Through the average translation $\bar{s}$ and rotation $\bar{q}$, the original position and orientation of each sensor are transformed to ${\bar{r^{\prime }}}_{m}$ and ${\bar{v^{\prime }}}_{m}$. The rotation and translation required for transforming the co-registered sensor positions $\left\{r_{m,n}^{\prime }|m=1,2,\ldots ,N_{c}\right\}$ and orientations $\left\{v_{m,n}^{\prime }|m=1,2,\ldots ,N_{c}\right\}$ of the *n*-th group to $\left\{{\bar{r^{\prime }}}_{m}|m=1,2,\ldots ,N_{c}\right\}$ and $\left\{{\bar{v^{\prime }}}_{m}|m=1,2,\ldots ,N_{c}\right\}$ are the translation error ${\dot{s}}_{n}$ and rotation error ${\dot{q}}_{n}$. The rotation error ${\dot{q}}_{n}$ and translation error ${\dot{s}}_{n}$ can be represented by the corresponding rotation angle θ_*n*_ and translation distance *d*_*n*_. For small angles, θ_*n*_ can be calculated by Sonntag et al. (2018)

(2) $$\begin{array}{c} \theta_{n}=2\,\sqrt{{\dot{q}}_{n,1}^{2}+{\dot{q}}_{n,2}^{2}+{\dot{q}}_{n,3}^{2}} \end{array}$$

and *d*_*n*_

(3) $$\begin{array}{c} d_{n}=\sqrt{{\dot{s}}_{n,1}^{2}+{\dot{s}}_{n,2}^{2}+{\dot{s}}_{n,3}^{2}} \end{array}$$

where ${\dot{q}}_{n,1}$, ${\dot{q}}_{n,2}$, and ${\dot{q}}_{n,3}$ are the imaginary parts of the quaternion ${\dot{q}}_{n}$ and ${\dot{s}}_{n,1}$, ${\dot{s}}_{n,2}$, and ${\dot{s}}_{n,3}$ are the scalar parts of ${\dot{s}}_{n}$. The repeatability errors of rotation and translation are calculated by the average rotation angle and translation distance:

(4) $$\begin{array}{c} \varepsilon_{r\,e\,p\,e\,a\,t\,\_\,r\,o\,t}=\frac{1}{N_{p}}\,\sum_{n=1}^{N_{p}}\theta_{n}\,\text{and}\,\varepsilon_{r\,e\,p\,e\,a\,t\,\_\,t\,r\,a\,n}=\frac{1}{N_{p}}\,\sum_{n=1}^{N_{p}}d_{n} \end{array}$$

Further, the repeatability errors of the co-registered sensor location, ε_*repeat*_*pos*_, and orientation, ε_*repeat*_*ori*_, can also be calculated (Pfeiffer et al., 2018, 2020)

(5) $$\begin{array}{c} \varepsilon_{r\,e\,p\,e\,a\,t\,\_\,p\,o\,s}=\frac{1}{N_{c}\times N_{p}}\,\sum_{m=1}^{N_{c}}\sum_{n=1}^{N_{p}}{\left|r_{m,n}^{\prime }-{\bar{r^{\prime }}}_{m}\right|}_{ℱ} \end{array}$$

and

(6) $$\begin{array}{c} \varepsilon_{r\,e\,p\,e\,a\,t\,\_\,o\,r\,i}=\frac{1}{N_{c}\times N_{p}}\,\sum_{m=1}^{N_{c}}\sum_{n=1}^{N_{p}}\arccos\left(v_{m,n}^{\prime }\cdot \frac{{\bar{v^{\prime }}}_{m}}{{\left|{\bar{v^{\prime }}}_{m}\right|}_{ℱ}}\right) \end{array}$$

where (.) denotes the dot product operator.

##### Final co-registration error

For the reference phantom, the sensor positions and orientations were known relative to MRI. Therefore, we could calculate the rotation and translation error between two coordinate systems where the co-registered sensor positions and the real sensor positions were separately located. The rotation and translation errors were considered as an index to quantify the final co-registration accuracy of each device. As previously mentioned, through the average translation $\bar{s}=\sum_{n=1}^{N_{p}}s_{n}/N_{p}$ and rotation $\bar{q}=\sum_{n=1}^{N_{p}}q_{n}\,/\,\left|\sum_{n=1}^{N_{p}}q_{n}\right|$, the original sensor positions and orientations were transformed to ${\bar{r^{\prime }}}_{m}$ and ${\bar{v^{\prime }}}_{m}$. The rotation and translation required for transforming ${\bar{r^{\prime }}}_{m}$ and ${\bar{v^{\prime }}}_{m}$ to the real sensor positions *r*_*m*_ and orientations *v*_*m*_ are the rotation error $\dot{q}$ and translation error $\dot{s}$. Thus, the final co-registration errors of the rotation and translation are calculated as

(7) $$\begin{array}{c} \varepsilon_{f\,i\,n\,a\,l\,\_\,r\,o\,t}=\theta \,\mathrm{and}\,\varepsilon_{f\,i\,n\,a\,l\,\_\,t\,r\,a\,n}=d \end{array}$$

where θ and *d* are the corresponding rotation angle and translation distance of $\dot{q}$ and $\dot{s}$, respectively, which could be calculated by (2) and (3). Further, the final co-registration errors of sensor positions and orientations can also be calculated by

$$\varepsilon_{f\,i\,n\,a\,l\,\_\,p\,o\,s}=\frac{1}{N_{c}}\,\sum_{m=1}^{N_{c}}{\left|{\bar{r^{\prime }}}_{m}-r_{m}\right|}_{ℱ}\,a\,n\,d\,\varepsilon_{f\,i\,n\,a\,l\,\_\,o\,r\,i}$$

(8) $$=\frac{1}{N_{c}}\sum_{m=1}^{N_{c}}arccos\left(\frac{{\bar{v^{\prime }}}_{m}}{{\left|{\bar{v^{\prime }}}_{m}\right|}_{ℱ}}.v_{m}\right)$$

Unlike in the reference phantom experiment, the sensor positions and orientations were unknown in the human experiment. However, the laser scanner showed the highest accuracy for co-registration, as described in the “Results” section. Thus, the co-registration results obtained by the laser scanner were used as the “true” sensor positions and orientations to intuitively compare the final co-registration accuracy of the Polhemus and the structured-light scanner. That is, the average translation and rotation vectors of five measurements for the laser scanner can be calculated. Through this average translation and rotation, the transformed sensor positions and orientations were considered as the true values of *r*_*m*_ and *v*_*n*_. The final co-registration errors of the Polhemus and the structured-light scanner were calculated using (7) and (8).

#### Source Localization Simulation

##### Data generation

The MRI data of the participant in the human experiment were used in this simulation. The co-registered positions and orientations of 85 sensors obtained from the laser scanner in the human experiment were regarded as the true sensor configuration in the simulation. Using this sensor configuration, simulated data were generated using the boundary element method (BEM) model (Fuchs et al., 2002), provided by the OpenMEEG (Gramfort et al., 2010) in the Fieldtrip (Oostenveld et al., 2011), as the forward model to simulate a more realistic situation. The BEM model had three layers segmented from MRI data using Freesurfer (Fischl, 2012), including the brain (0.33 S/m), scalp (0.33 S/m), and skull (0.0041 S/m). The cortical surface of the participant was downsampled to a mesh with 1,000 vertices. In each dataset, one source was activated. The source waveform was simulated as a sinusoid with a frequency of 15 Hz and a source amplitude of 10 nA⋅m. The source position was at the vertex of the downsampled cortical surface, while the source orientation was restricted to be perpendicular to the local cortical surface (Lin et al., 2006). The simulated data were noise free. All sources at the downsampled cortical surface were simulated to obtain the simulated datasets.

##### Source localization

For source localization, the dipole-fitting method (Lütkenhöner, 1998) was used to solve the inverse problem. In the simulation, we analyzed the influence of model and co-registration errors on the source localization accuracy. We used two types of head models, the Nolte corrected-sphere model (Nolte, 2003) and the BEM model, to calculate the lead field needed to solve the inverse problem. To simulate the actual co-registration error of different devices, the co-registration results of the three devices in the human experiment were used as the sensor configuration when solving the inverse problem. The source localization error was calculated as the average of the Euclidean distances from the simulated source and reconstructed positions among all 1,000 simulated datasets.

## Results

### Reference Phantom Experiment

In the reference phantom experiment, five groups of data for each device were used for co-registration with pseudo MRI. The surface fit error, repeatability error, and final co-registration error were calculated to evaluate the co-registration performance of the devices.

Except for the surface fit error of Polhemus for transform 1, which is accomplished by aligning six reference points, the other surface fit errors are shown in Table 2. The surface fit error showed that the ICP fits finished with reasonable quality. The laser scanner had the smallest surface fit error and standard deviation in transforms 1 and 2.

**TABLE 2:** Surface fit error of each device in the reference phantom experiment.

**Device**	**Transform 1**	**Transform 2**
Polhemus	None	0.58 ± 0.41 mm
Structured-light scanner	2.08 ± 1.10 mm	0.49 ± 0.36 mm
Laser scanner	0.37 ± 0.18 mm	0.24 ± 0.10 mm

Use of the reference phantom allowed quantification of the final co-registration error. The final co-registration and repeatability errors of each device in the reference phantom experiment are shown in Table 3. The final co-registration errors of the laser scanner are a rotation error of 0.23°, a translation error of 0.76 mm, a corresponding sensor location error of <1 mm, and an orientation error of ∼0.2°, indicating excellent performance. Furthermore, the laser scanner had the smallest repeatability error, showing high consistency for multiple measurements. The co-registration accuracy of Polhemus was significantly better than that of the structured-light scanner, while its repeatability error was the worst.

**TABLE 3:** Final co-registration and the repeatability errors of each device in the reference phantom experiment.

**Device**	**Final co-registration error**				**Repeatability error**			
	**Rotation**	**Translation**	**Location**	**Orientation**	**Rotation**	**Translation**	**Location**	**Orientation**
Polhemus	0.37°	0.86 mm	1.22 ± 0.30 mm	0.27° ± 0.09°	0.54° ± 0.37°	1.08 ± 0.79 mm	1.32 ± 1.02 mm	0.44° ± 0.35°
Structured-light scanner	1.10°	1.52 mm	2.19 ± 1.02 mm	0.91° ± 0.24°	0.41° ± 0.41°	0.64 ± 0.31 mm	0.99 ± 0.74 mm	0.32° ± 0.30°
Laser scanner	0.23°	0.76 mm	0.72 ± 0.19 mm	0.18° ± 0.05°	0.06° ± 0.02°	0.06 ± 0.04 mm	0.10 ± 0.05 mm	0.04° ± 0.02°

In the phantom experiment, the co-registration accuracy was in the order of laser scanner > Polhemus > structured-light scanner. For the measurement, the repeatability accuracy was in the order of laser scanner > structured-light scanner > Polhemus.

### Human Experiment

The surface fit errors of the three devices in the human experiment are shown in Table 4. The surface fit error was the smallest when the laser scanner was used for co-registration. Compared to the results of the reference phantom experiment, the surface fit errors of transform 2 for all devices were increased. As shown in Table 5, the repeatability accuracy was still in the order of laser scanner > structured-light scanner > Polhemus.

**TABLE 4:** Surface fit error of each device in the human experiment.

**Device**	**Transform 1**	**Transform 2**
Polhemus	None	2.16 ± 0.97 mm
Structured-light scanner	2.19 ± 1.18 mm	1.64 ± 0.78 mm
Laser scanner	0.53 ± 0.46 mm	1.56 ± 0.66 mm
		

**TABLE 5:** Repeatability error of each device in the human experiment.

**Device**	**Rotation**	**Translation**	**Location**	**Orientation**
Polhemus	1.20° ± 0.25°	1.34 ± 0.58 mm	1.96 ± 0.75 mm	0.92° ± 0.48°
Structured-light scanner	0.77° ± 0.16°	1.10 ± 0.49 mm	1.70 ± 0.71 mm	0.58° ± 0.13°
Laser scanner	0.14° ± 0.04°	0.21 ± 0.11 mm	0.30 ± 0.11 mm	0.11° ± 0.03°
				

In the human experiment, the true positions and orientations were unknown. As the laser scanner showed the best performance, we used average translation and rotation to transform the original sensor positions and orientations to the co-registered results and considered these results as the pseudo “ground truth” for sensor positions and orientations to compare the co-registration errors of the other two devices. Using the laser scanner as the reference, the final co-registration errors of Polhemus and structured-light scanner (Table 6) showed higher accuracy for the structured-light scanner, contrary to the results of the phantom experiment. This unexpected result may be due to the following two reasons. First, the phantom remained stationary in the reference phantom study, while the participant in the human experiment could not remain completely immobile. Second, if the structured-light scanner and laser scanner were more similar to each other than to Polhemus, the use of the laser scanner as a ground truth may have biased the comparison. The results of two supplementary experiments (Supplementary Tables 1, 2) indicated that both the participant movement and the similarity between two optical scanners affected the comparison of Polhemus and the structured-light scanner. However, the movement had a greater influence on the co-registration accuracy of Polhemus.

**TABLE 6:** Final co-registration error of each device in the human experiment.

**Device**	**Rotation**	**Translation**	**Location**	**Orientation**
Polhemus	1.39°	3.12 mm	3.47 ± 1.41 mm	1.12° ± 0.27°
Structured-light scanner	1.32°	1.30 mm	1.96 ± 0.73 mm	1.06° ± 0.28°
				

### Simulation Results

Table 7 shows the source localization errors for different devices and head models. When the co-registered positions and orientations of the laser scanner were used as the sensor configuration (i.e., there was no co-registration error), the source localization error was 0.23 mm under the BEM model, reflecting the error caused by the inverse solution. When the sensor configuration was changed, Polhemus (co-registration location error: 3.47 ± 1.41 mm, orientation error: 1.12 ± 0.27°) showed a higher source localization error due to its higher co-registration error than that for the structured-light scanner.

**TABLE 7:** Source localization error using different co-registration methods and head models.

**Device**	**BEM model**	**Nolte corrected-sphere model**
Laser scanner	0.23 ± 1.28 mm	1.06 ± 1.80 mm
Polhemus	3.54 ± 1.37 mm	3.86 ± 1.85 mm
Structured-light scanner	1.44 ± 1.24 mm	1.90 ± 1.82 mm

In clinical practice, it is impossible to build a completely correct head model to describe the relationship between the sources in the brain and sensors. Therefore, we also simulated situations with head model error. The Nolte corrected-sphere head model resulted in increased source localization error. When the sensor configuration was obtained from Polhemus, changing the head model increased the source localization error by 9%. Under the higher co-registration error (Polhemus level), the source localization error caused by the inverse solution and the head model was relatively small.

## Discussion

This study reviewed the existing devices and relative methods used for the co-registration of on-scalp MEG and MRI. A reference phantom was constructed to provide the ground truth of sensor positions and orientations, making it possible to calculate the true co-registration error. The co-registration errors of each device were quantified and compared in the reference phantom and human experiments.

Previous studies have reported optimal co-registration errors of on-scalp MEG sensor positions of <4 mm and orientation errors of <10° to obtain similar or higher localization accuracy to that of SQUID-MEG (Zetter et al., 2018). The results of the reference phantom experiment in the present study showed that the three types of devices met these requirements; in particular, the sensor orientation errors were much smaller than the theoretical requirements. The results of the source localization simulations revealed the serious impact of low co-registration accuracy on the source localization compared to the inverse solution and head model errors. High co-registration accuracy is the premise for further improving the source localization accuracy. The laser scanner showed the best accuracy and precision among the three devices. Therefore, it is the best choice for high co-registration requirements. For practical applications, unless the participant can be immobilized during digitization, the effect of motion on Polhemus makes its performance inferior to that of the structured-light scanner. In addition, the structured-light scanner showed the best cost performance and was more convenient to use. Thus, the structured-light scanner is recommended over Polhemus.

Due to the lack of the information on the true sensor positions and orientations in clinical practice, previous studies mainly evaluated the co-registration error through surface fit or repeatability errors (Zetter et al., 2019; Hill et al., 2020; Gu et al., 2021). The present study considered the final co-registration error as the standard to quantify the co-registration. Compared to the final co-registration error of sensor positions and orientations, the transformation parameter errors (rotation and translation) can be used to compare the co-registration results of using different sizes and shapes of the helmet. In practical applications, the final co-registration error cannot be obtained. Researchers can monitor the co-registration performance of the used device based on the repeatability error and the target registration error (TRE) (Sonntag et al., 2018). The repeatability error can be a measure of co-registration precision. TRE resulting from the uncertainty of the co-registration can be used to evaluate the co-registration. Considering both accuracy and precision, average rotations and translations for multiple measurements to obtain the final transformation parameters are recommended to improve the co-registration.

Researchers should consider some precautions when using the device for practical applications. Generally, digitization or scanning should be performed before acquiring MEG measurements or when the sensors are removed after the experiment. In addition, when using Polhemus for co-registration, researchers should be aware of the following: (1) electromagnetic interference must be eliminated since it affects the performance of Polhemus; (2) the reference receiver must be used; (3) the participant should keep as still as possible. When using the structured-light scanner, the scanning should be performed in a well-lit environment and with good network conditions to guarantee high-quality data transfer. The laser scanner has no environmental requirements.

For on-scalp MEG, sensors should be placed as close as possible to the scalp of the participant. Especially for OPM-based MEG, the depth of the sensors can be adjusted freely according to the distance between the rigid helmet and the scalp. The present study focused on the comparison of the co-registration performance of three devices and did not consider the depth information. However, practical applications require that the depths of sensors be recorded. Following the co-registration of each device, the positions of the sensors must be corrected according to their recorded depths. The accuracy of the depth will influence the accuracy of the sensor location. Although the use of a high-precision vernier caliper allows measurement of depth at an accuracy of 0.1 mm or better, the trend of increasing numbers of sensors for on-scalp MEG (Tierney et al., 2019) makes the use of a vernier caliper to measure each sensor depth time-consuming. The development of fast and precise methods for depth measurement will benefit the practical applications of OPM-based MEG using the rigid helmet.

## Conclusion

The results of this study present a detailed and comprehensive comparison of the co-registration accuracies of the three devices currently used for the co-registration of on-scalp MEG and MRI. We have proposed a reference phantom and considered the final co-registration errors as the standard indices to quantify the co-registration performance of each device. Higher co-registration accuracy is needed to achieve the optimal performance of the on-scalp MEG. The laser scanner is the best choice since it showed the lowest co-registration error. The structured-light scanner is recommended over the Polhemus because it is less influenced by the participant movement in practical applications and has the best cost performance.

## Data Availability Statement

The raw data supporting the conclusions of this article will be made available by the authors, without undue reservation.

## Ethics Statement

The studies involving human participants were reviewed and approved by Beihang University Ethical Committee. The patients/participants provided their written informed consent to participate in this study. Written informed consent was obtained from the individual(s) for the publication of any potentially identifiable images or data included in this article.

## Author Contributions

FC conceived and designed the study and wrote the first draft of the manuscript. WX, WW, and YY performed the experiments. FC and NA analyzed the data. XN, MX, and YG supervised the research. XN and NA reviewed and edited the manuscript. All authors read and approved the submitted version.

## Conflict of Interest

The authors declare that the research was conducted in the absence of any commercial or financial relationships that could be construed as a potential conflict of interest.

## Publisher’s Note

All claims expressed in this article are solely those of the authors and do not necessarily represent those of their affiliated organizations, or those of the publisher, the editors and the reviewers. Any product that may be evaluated in this article, or claim that may be made by its manufacturer, is not guaranteed or endorsed by the publisher.

## Footnotes

1
http://www.uniontech3d.cn/product/detail/1679

2
https://polhemus.com/_assets/img/FASTRAK_Digitizer_Brochure.pdf

3
https://support.structure.io/article/377-how-precise-is-structure-sensor- mark-ii

4
https://www.sikantech.com/products_434.html

5
https://skanect.occipital.com/
