# Supplementary material for: Co-registration Comparison of On-Scalp Magnetoencephalography and Magnetic Resonance Imaging
Source: Front Neurosci. 2021 Aug 13;15:706785. doi: 10.3389/fnins.2021.706785 (PMC8414551; doi:10.3389/fnins.2021.706785)
Supplement: Supplementary file 1 [file Data_Sheet_1.docx]

Supplementary Material

Polhemus showed better performance than the structured-light scanner in the reference phantom experiment, contrary to the results in the human experiment. To further analyze the causes of this phenomenon, we have added two supplementary experiments to illustrate it.

# Supplementary Experiment 1

One cause is that in the reference phantom experiment, the phantom remained stationary, while in the human experiment, the participant could not remain immobile at all times. To analyze the influence of the small displacement of the participant on the co-registration performance of three devices, a supplementary experiment was conducted. The experiment was performed five times using three devices in the two states of the reference model: being stationary and being held by the participant. The small motion of the reference model was simulated by the participant holding the reference model. The participant was also asked to remain as still as possible. We calculated the final co-registration location error of each device, as shown in Table S1. The results showed that the final co-registration location error from static to motion increased 0.88 mm for Polhemus, 0.27 mm for the structured-light scanner and 0.06 mm for the laser scanner. Motion affects more on the Polhemus than the other two optical scanners.

For Polhemus, although the use of the reference receiver can diminish the influence of the displacement, its correction is limited. The two optical scanners are always displaced relative to the scanned object when used. The scanners can automatically track the movement of the object through features provided by a larger number of points, which is more efficient in providing motion compensation compared to Polhemus.

**Table S1** Final co-registration location error of each device in the static and small motion state of the reference phantom

| Device | Static | Motion |
| --- | --- | --- |
| Polhemus | 1.40 mm | 2.28 mm |
| Structured-light scanner | 2.08 mm | 2.35 mm |
| Laser scanner | 0.67 mm | 0.73 mm |

# Supplementary Experiment 2

The other cause may be that the structured-light scanner and laser scanner were more similar to each other than to Polhemus. Therefore, the use of the laser scanner as a ground truth may bias the comparison of the structured-light scanner and Polhemus. To analyze this similarity whether influence the comparison, a supplementary experiment was conducted.

We used the co-registration results of the laser scanner in the phantom experiment as the “ground truth” of sensor positions and orientations to compare the co-registration error of Polhemus and the structured-light scanner. The results revealed that the final co-registration location error of Polhemus decreased 0.06 mm when the reference changed from the ground truth to the results of the laser scanner, while that for the structured-light scanner decreased by 0.21 mm.

**Table S2** Final co-registration location error of Polhemus and the structured-light scanner when use the ground truth or the co-registration results of the laser scanner as the reference

| Device | Ground truth as the reference | Laser scanner as the reference |
| --- | --- | --- |
| Polhemus | 1.22 mm | 1.16 mm |
| Structured-light scanner | 2.19 mm | 1.98 mm |
